# On the Meaning of the Term “Ulceration,” as at Present Applied to Uterine Diseases

**Published:** 1852-07

**Authors:** T. Snow Beck

**Affiliations:** Physician to the Farrington General Dispensary and Lying-in Charity


*On the meaning of the term "Ulceration," as at present applied to uterine diseases.* By T. Snow Beck, M. D., F. R. S., Physician to the Farrington General Dispensary and Lying-in Charity.—The conflicting opinions which have been expressed by practitioners as to the absence or presence of ulceration at the orifice of the uterus, appear only to be accounted for by referring to the different senses in which this word has been used. At first sight it appears strange, that two opinions should exist as to the meaning of a word the definition of which is so generally agreed upon; yet it is, nevertheless, certain, that in regard to the diseases of the uterus, it has been employed to denote morbid conditions which are very different from ulcerations, in the received acceptation of the term.

The definition given by all surgeons is essentially the same. Thus, for example, Boyer states: "An ulceration is a solution of continuity of the soft parts, more or less ancient, accompanied by a purulent secretion, and kept up by some local or internal cause;" and Samuel Cooper says: "Surgeons usually define an ulcer to be a solution of continuity in any of the soft parts of the body, attended with a secretion of pus, or some kind of discharge." Authors on practical medicine and pathology likewise adopt a similar definition. Dr. Copland considers "ulceration is distinguished from suppuration chiefly by its being attended by a loss of substance, by a destruction of parts, and by a more or less abundant secretion of puriform, ichorous, fetid, sanious, or variously-colored fluid." Dr. Watson writes: "We may say, with Dr. Allison, that, whenever the absorption of the effused lymph, and of the surrounding textures, takes place in excess—in a greater degree, that is, and more irregularly than seems to be required for any useful purpose—the result is *ulceration*. The term is, however, commonly restricted to those cases in which the loss of substance occurs upon some *surface*, internal or external." Dr. Williams defines an ulcer to be "a solution of continuity or excavation, the bottom and edges of which continue to discharge pus, or a serous fluid mixed with exudation corpuscles, and sometimes blood corpuscles." Chomel states, that "ulcers are solutions of continuity maintained (*entre tenues*) by an internal or local disease." Andral says, "The term ulcer is applied to that solution of continuity which is produced in a tissue by the absorption of its molecules."

According to these definitions, to which many more might be added, the essential character of ulceration is a solution of continuity or excavation, or a loss of substance in a part; for it is evident, that the discharges which usually accompany this lesion are not characteristic of, nor essential to it, inasmuch as they are also products of simple inflammation of a mucous membrane. The accuracy of these definitions are confirmed by more recent observation of morbid phenomena by the aid of the microscope, which has shown, that, previous to ulceration taking place, the blood becomes stagnant, and adheres to the walls of the vessels, that the neighboring tissues thus lose their vitality, by changes similar to what occur in mortification, and, being carried away by effused fluid, a solution of continuity, or loss of substance, is produced. To express this series of morbid changes in a short phrase, many pathologists define ulceration as "molecular death," or "molecular gangrene." But this does not in any way alter the essential character of the previous definitions: for, when "molecular death or gangrene" occurs in any tissue, "a solution of continuity, or loss of substance" in that tissue is the inevitable result.

Admitting, then that these definitions correctly express the essential character of ulceration, we may inquire, whether this term has been applied with propriety to many morbid states of the orifice of the uterus. At the late discussion at the Royal Medical and Chirurgical Society, Dr. Murphy is reported to have said: "A girl was admitted into the hospital (University College) dying from the effects of opium. At the autopsy, he had examined the uterus, and found an ulcer on both lips extending into the cavity. It was seen by the pupils, and was sent to the curator to be preserved, but the next day it had lost all its characters, and was thrown aside." With all respect for Dr. Murphy's high standing in the Profession, and the personal esteem which must be felt by all who know him personally, I can only come to the conclusion, that some source of error must exist in this statement. A solution of continuity, or a loss of substance, on the lips of the uterus, would have been as readily recognised the day after the autopsy as on the day of the examination; and, if the supposed ulceration "had lost all its characters the next day," is it not certain that ulceration had not existed, and that some other morbid appearance had been mistaken for it? How could the loss of substance be repaired? And, if still present, it would have been recognised. I feel the greater confidence in speaking upon this subject, since I have carefully searched during some years for these reputed ulcerations in my own practice, and in the hospital practice of other physicians, without being enabled to discover them. During this period, I have seen many cases of inflammation of the mucous membrane covering the lips of the uterus, and even extending into the cervical canal, which presented a red and granular appearance, and which had been designated ulceration, but wherein no loss of substance or solution of continuity existed. Sometimes the glands in the mucous membrane were chiefly implicated in the inflammation, and each being surrounded by a defined circle of redness, they presented, when grouped together, a circumscribed and defined granular patch, studded with minute pits or holes, which led to the orifices of the glands. In these cases, the pathological condition did not differ from that of chronic inflammation in other mucous membranes, as, for example, in the granular condition of the conjunctiva; in the state of the mucous membrane of the stomach, to which the name of mammillation has been applied, in the condition sometimes observed in the urethra of the male after gonorrhœa, etc. In these instances, there is no loss of substance in the membranes, nor has any one contended that ulceration was present; yet this name has been applied to the same morbid conditions when observed in the mucous membrane of the vagina, or in that covering the lips of the uterus. With this explanation, it can readily be understood, that an inflamed mucous membrane might lose its characteristic appearances the day after the autopsy, especially if it had been immersed in any fluid; but if a loss of substance or an ulcer had existed, that ulcer would have been as readily discovered the next day, as on the day on which the first examination was made.

At the same meeting of the Society, a letter was read from Mr. Holl, detailing the result of his *post-mortem* examinations, and bearing upon this subject. Dr. H. Bennett afterwards remarked: "That it appeared to him that Mr. Holl's guarantee for the correctness of his opinions was all that could be desired, as he was practically acquainted with the lesions to which that part is subject in the living state; that he had carefully examined the condition of the parts, and stated in his letter,—'The whole number of bodies in which the state of the uterus was examined was 44; of these 34 either presented no disease of the cervix uteri, or only slight congestion, with the exception of 3, in whom the congestion was considerable. Of the remaining 10 cases, 9 of them presented more or less extensive abrasion of the epithelium from around the os uteri, but no ulceration. The pelvic veins were generally full of blood; the cervix uteri large, swollen, and puffy, with more or less venous congestion of the mucous and sub-mucous tissues, but no induration or effusion of lymph into its texture. The glands of Naboth were enlarged, and the canal of the cervix blocked up with viscid mucus. The removal of the epithelium appeared to commence at the follicles which surrounded the cervix uteri." Dr. Bennett, having read the letter, drew attention to the fact, that Mr. Holl, having a knowledge of these special lesions, and having specially investigated them in the autopsies, discovered 10 cases out of 44, or 37 per cent., in which there existed abrasion, or ulceration of the uterine mucous membrane,—conditions which he (Dr. Bennet) considered pathologically identical. This, however, appears a perversion of Mr. Holl's words, who distinctly says, "there was abrasion of the epithelium, *but no ulceration.*" After lauding Mr. Holl's knowledge of these special lesions as offering a guarantee,—all that could be desired for the correctness of his opinions,—it appears a singular proceeding to ignore his knowledge, and contradict the opinion founded upon it. Would it not have been more consonant with the facts to have stated, Mr. Holl said he had found nine cases of abrasion of the epithelium, but no ulceration; yet this statement was erroneous, as, in reality, there were cases of ulceration? And that this conclusion was come to, in opposition to the former praise, because abrasion of the epithelium and ulceration were considered conditions pathologically identical. But is this the fact? Are these lesions pathologically identical?

The essential character of ulceration—that it is a solution of continuity or loss of substance in a tissue—has been sufficiently proved, and the pathological changes which occur have also been pointed out. In abrasion, or excoriation, the epithelial covering of the mucous membrane is removed, but the substance of the membrane itself remains uninjured. There is no solution of continuity, or excavation, or loss of substance in the part, and this constitutes the essential difference between the two lesions—abrasion, or excoriation, and ulceration. The pathological changes which occur are equally distinct. In ulceration, the blood becomes stagnant, adheres to the sides of the vessels, the vitality of the part is destroyed, molecular death or gangrene being induced. In excoriation, the blood flows on without interruption or stagnation; a portion of serum is effused beneath the unorganized epithelium, which, being detached, is subsequently removed. The changes which occur may be compared to the effect of an exceedingly minute and mild blister, active in its character, acute in its course; whilst ulceration is the consequence of minute mortification, usually slow in its action, chronic in its course. The difference between the two lesions, and the pathological changes which occasion them, thus appear to be well defined; but there is one point of view from which the two might be confounded. The first change occurring in a mucous membrane about to undergo minute gangrene is, in some cases, the effusion of bloody serum beneath the epithelium, and the subsequent desquamation of this part. And if we were to inquire no further into the changes going on, it might be concluded that, as there is desquamation of the epithelium in the beginning of the process of ulceration, and also in excoriation, that hence the two states are pathologically identical. But this would be a very superficial mode of considering the question. In the changes which produce ulceration, desquamation of the epithelium is not the only alteration which takes place in the membrane; for, after its removal, the surface appears livid and dark-colored, the tissues having lost their vitality, and the vessels of the part being blocked up, as in mortification. But in excoriation, the separation of the epithelium is the first and only change which occurs in the tissues of the membrane, which, after its removal, appears red and florid, the vessels being pervious, and the blood circulating through them. Excoriations, then, are the consequence of inflammation of the mucous membrane, or, as some have expressed, of catarrhal inflammation, more or less acute; ulcerations are the results of molecular gangrene, or mortification, the sequence of disease extending into the deeper tissues of the organ. Thus, even from this point of view, it would be illogical and erroneous to consider these two morbid conditions as pathologically identified.

The importance of this subject is greatly increased when considered in relation to the treatment of the diseases of the uterus. From many communications I have received, I have no doubt that the idea of having so loathsome a disease as "ulceration" on the uterus, has induced many females to submit to the employment of the speculum, and escharotics; while the same term has been considered by practitioners as a justification for this mode of treatment. It is thus intimately connected with the proper or improper use of the speculum, in the treatment of uterine disease. Where ulceration really exists, which is very rare, unless it be specific ulceration, the local application of escharotics by the aid of the speculum, during the later stages of the disease, is, no doubt, an useful adjunct to the other means of cure; but when excoriation only is present, or a reddened and swollen state of the mucous membrane, conditions which accompany inflammation more or less acute, the employment of these therapeutical agents is not only improper, but frequently most injurious.

It appears to have been overlooked in the discussions on this subject, that neither excoriations nor ulcerations are, in themselves, the essential disease; but that they are merely symptoms or evidences of the existence of other diseases which have produced these morbid states. This is well shown in Mr. Holl's account of the morbid appearance, where "the pelvic veins were generally full of blood, the cervix uteri large, swollen, and puffy, with more or less venous congestion of the mucous and submucous tissues, but no induration or effusion of lymph into its texture. The glands of Naboth were enlarged, and the canal of the cervix blocked up with viscid mucus,"—distinct evidences of inflammation of the substance of the organ,—while "the removal of the epithelium, which appeared to commence at the follicles surrounding the cervix uteri," was but a sign of this inflammation affecting the glands imbedded in the tissues of the part. Viewed in this light the application of escharotics to remove a symptom, whilst the disease producing it is unheeded, is undoubtedly not the correct course to pursue; nor can it be correct to endeavor to remove the symptom of acute inflammation of the mucous membrane—excoriation—by the application of substances which increase the inflammation already present. This mode of designating a reddened and swollen mucous membrane an ulceration, and including in one group diseases which are pathologically distinct, and which require opposite methods of treatment, has been productive of a serious amount of mischief in the treatment of uterine affections. Thus, in subacute inflammation of the mucous membrane of the vagina, where the membrane is red and swollen, and where the symptoms were, pain deep in the hypogastrium, pain on passing the fæces or urine, a mucous or purulent vaginal discharge, with pain across the forehead, and other constitutional symptoms, I have known the speculum employed, and the nitrate of silver applied, in consequence of the practitioner being misled by the idea that extensive ulceration existed. As might be supposed, this treatment was followed by great increase in the symptoms for several subsequent days, without any permanent advantage. On the other hand, in some exceptional cases of chronic vaginitis, which chiefly involves the upper part of the vagina, and where lotions have been inefficiently employed, either from inattention or want of management on the part of the female, the local application of strong stimulants has altered the morbid action, removed the thickened or mammilated condition of the membrane, subdued the vaginal discharges, and other symptoms. But these are only exceptional cases, and, by no means form the general rule. Similar errors have been committed in respect to the diseases of the uterus itself. In cases of inflammation of the organ, the redness of one of the lips, or the red appearance of the lining mucous membrane, have been treated by the application of escharotics, and followed by much augmentation of the inflammatory action in the deeper tissues. Even in those cases where ulceration, or a loss of substance, has really existed, much injury results from concentrating the attention too much upon this lesion, and neglecting the diseases or morbid states to which it owes its origin. What surgeon would think of applying powerful local applications to an ulcer on the leg, in the expectation of curing it, until he had first removed the state of the venous circulation upon which the continuance of the ulcer depends? Yet this is exactly what has been continually done in the treatment of diseases of the uterus.

These principles were well expressed by Professor Dubois, during the discussion before the Academy of Medicine at Paris, in 1850: "The value which has been given by one of our colleagues to ulcerations, if this were real, leads to consider them as one of the local conditions the most worthy of attention in practice. I cannot, for my part, adopt this manner of view; according to my appreciation, these lesions, as well as "engorgements," are only consequetive phenomena of another alteration (inflammation) which has preceded them, and have in general in regard to the symptoms and treatment, but a secondary importance."—*Bulletin.* *London Lancet.*

